# Supplementary material for: Sustained enzymatic activity and flow in crowded protein droplets
Source: Nat Commun. 2021 Nov 1;12:6293. doi: 10.1038/s41467-021-26532-0 (PMC8560906; doi:10.1038/s41467-021-26532-0)
Supplement: Supplementary file 4 — Supplementary Data 1 [file 41467_2021_26532_MOESM4_ESM.pdf]

Appendix: NMR Spectra

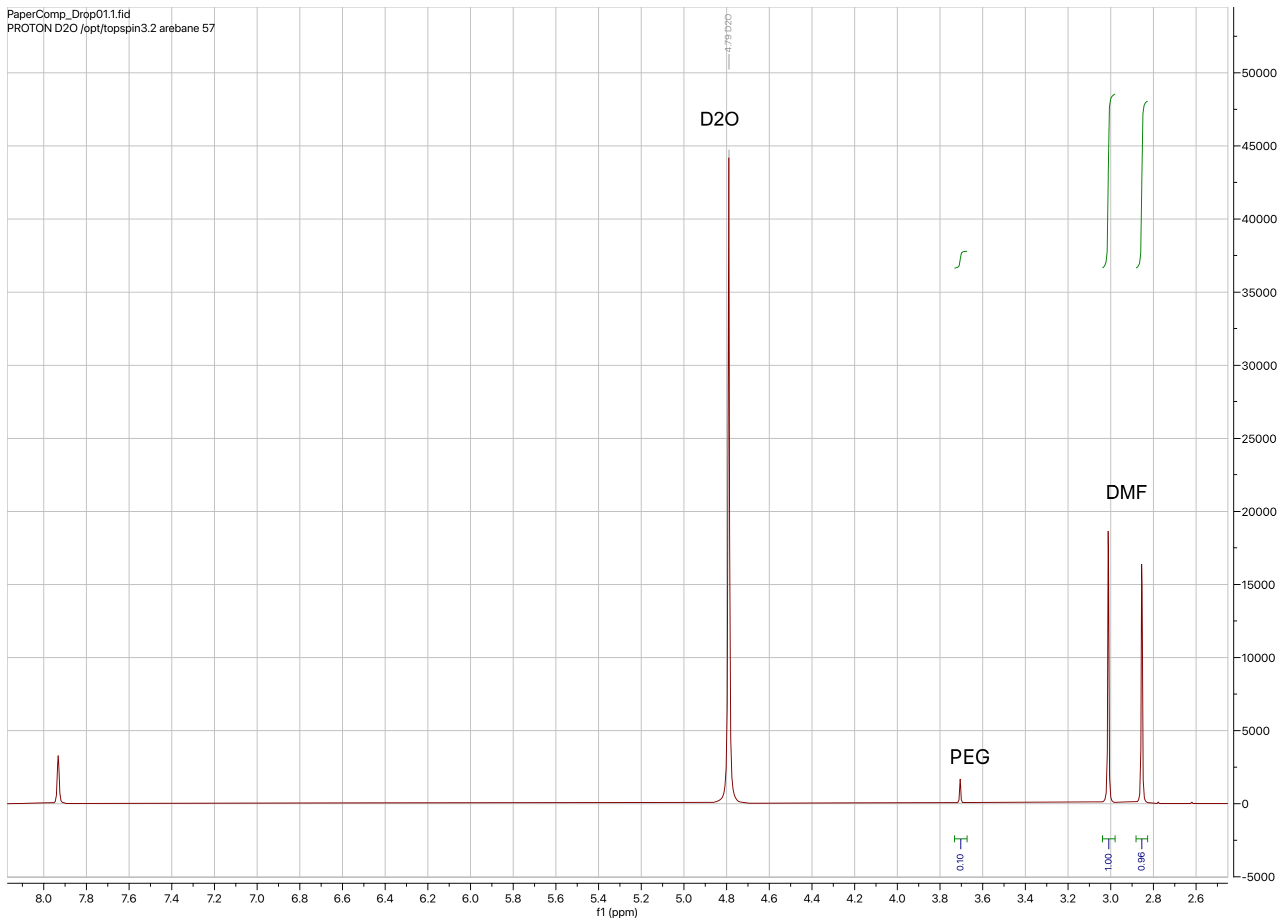

FIG. 1. **Droplet:** Global snapshot of ^{1}H NMR Spectrum of droplet phase under typical working conditions, with DMF used as concentration standard. The solvent and PEG peaks are labelled as D2O and PEG, respectively. The two rightmost peaks correspond to a total of six protons from the two methyl groups of DMF. The leftmost peak corresponds to the formyl proton on DMF, which is not considered during the PEG concentration measurement.

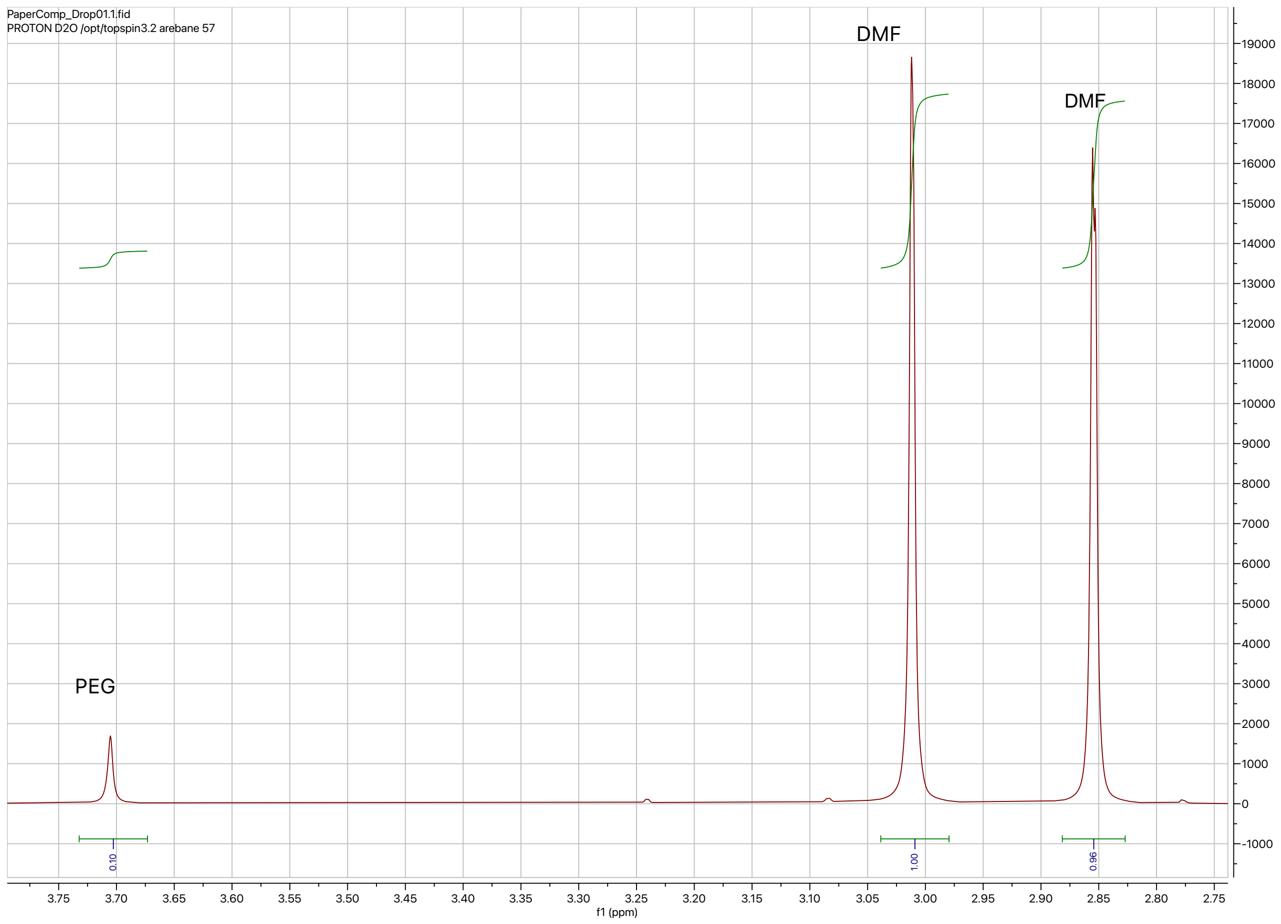

FIG. 2. **Droplet (magnified):** ^{1}H NMR Spectrum shown in FIG. 1 scaled to fully resolve the PEG and DMF peaks used in the PEG concentration measurement.

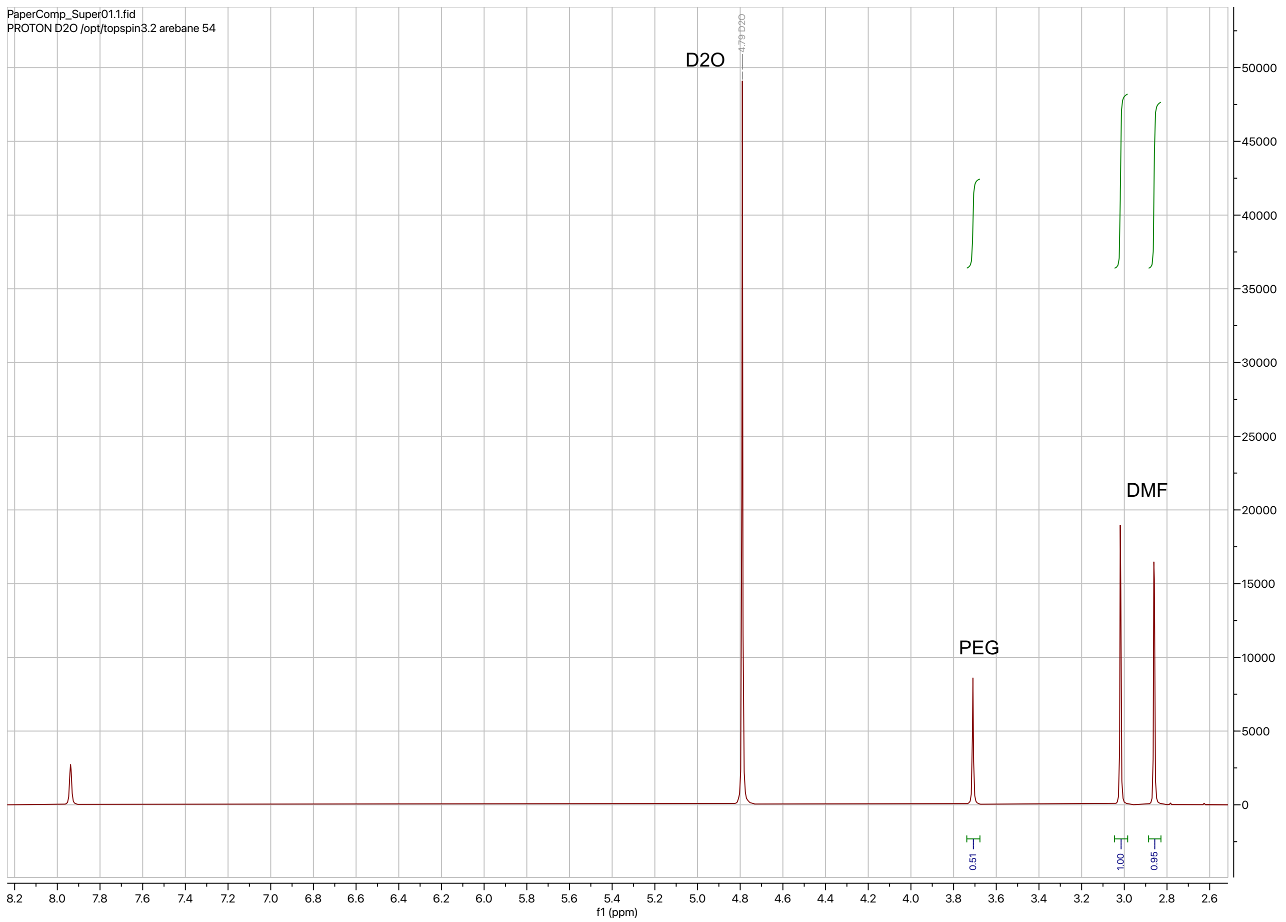

FIG. 3. **Supernatant:**
Global snapshot of ^{1}H NMR Spectrum of supernatant phase under typical working conditions, with DMF used as concentration standard. The solvent and PEG peaks are labelled as D2O and PEG, respectively. The two rightmost peaks correspond to a total of six protons from the two methyl groups of DMF. The leftmost peak corresponds to the formyl proton on DMF, which is not considered during the PEG concentration measurement.

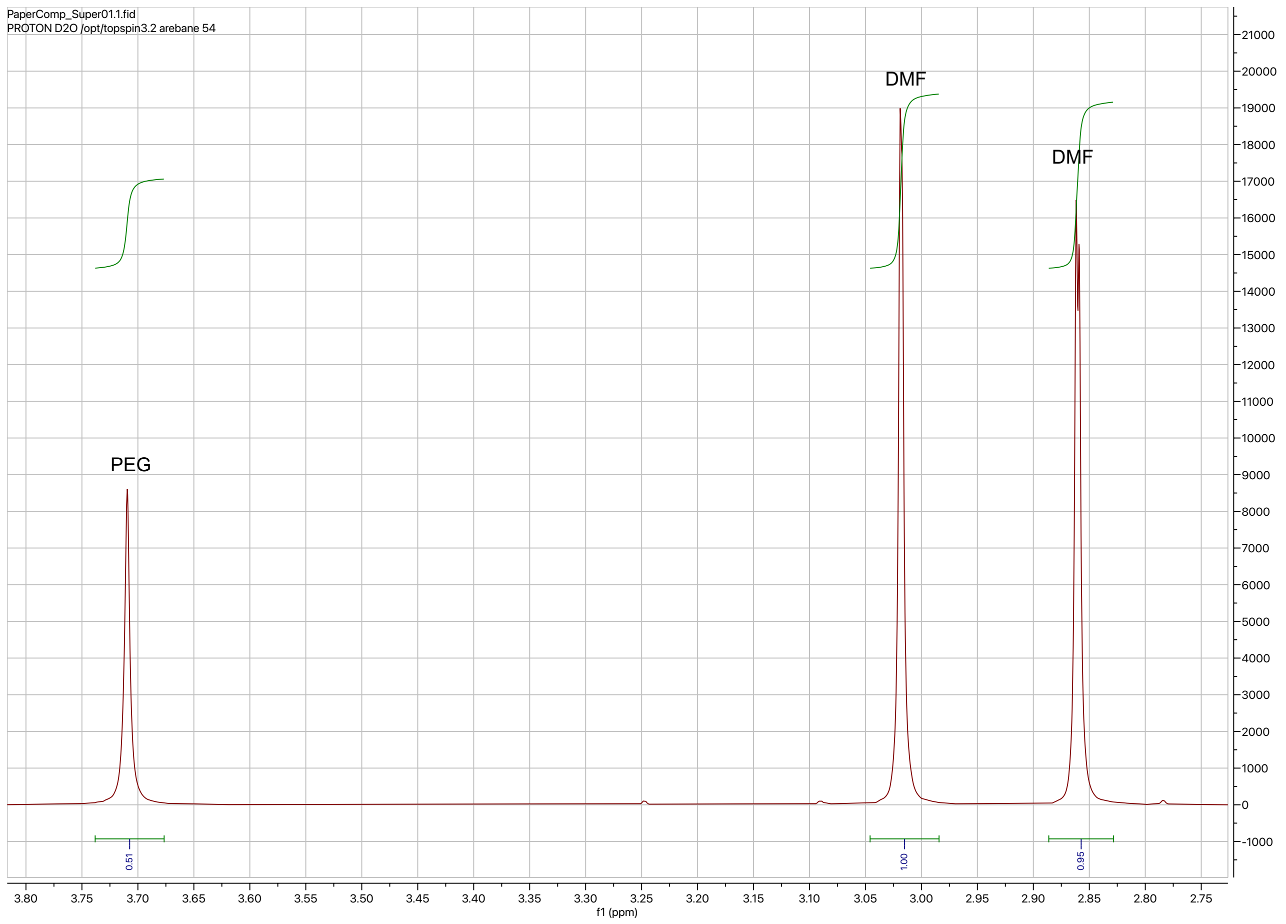

FIG. 4. **Supernatant (magnified):** ^{1}H NMR Spectrum shown in FIG. 3 scaled to fully resolve the PEG and DMF peaks used in the PEG concentration measurement.

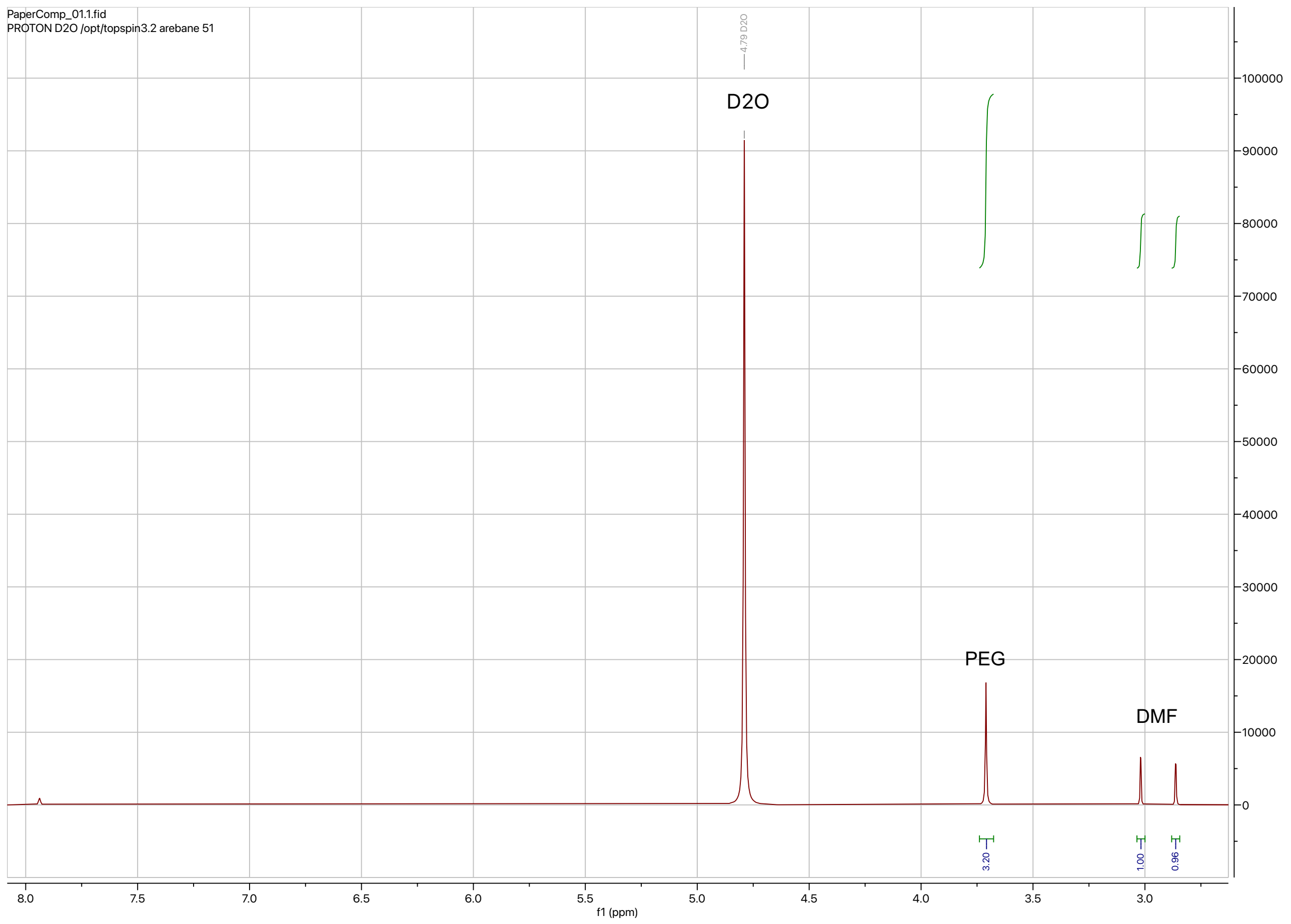

FIG. 5. **Total composition:**

Global snapshot of ^{1}H NMR Spectrum of droplet suspension under typical working conditions, with DMF used as concentration standard. The solvent and PEG peaks are labelled as D2O and PEG, respectively. The two rightmost peaks correspond to a total of six protons from the two methyl groups of DMF. The leftmost peak corresponds to the formyl proton on DMF, which is not considered during the PEG concentration measurement.

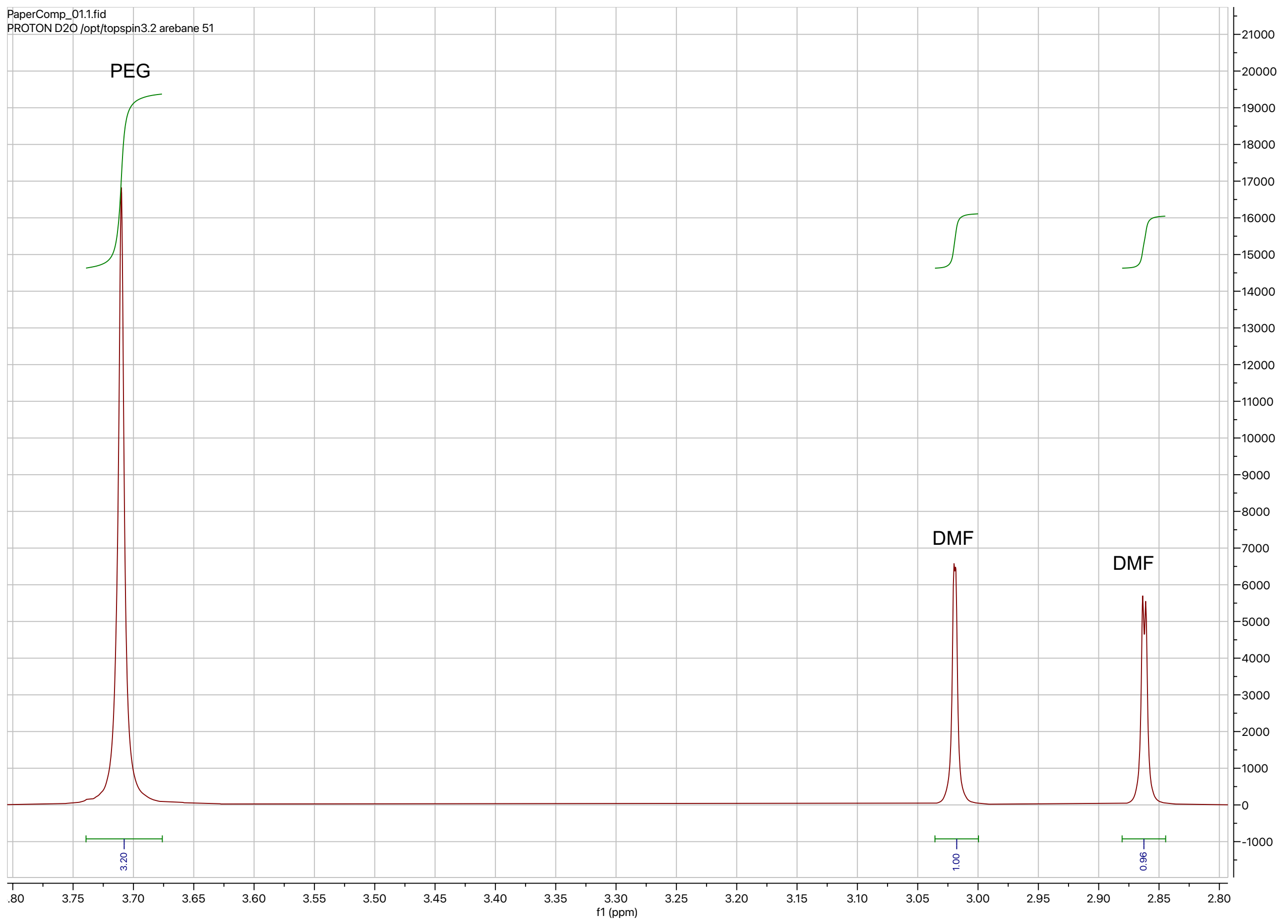

FIG. 6. **Total composition:** ^{1}H NMR Spectrum shown in FIG. 6 scaled to fully resolve the PEG and DMF peaks used in the PEG concentration measurement.

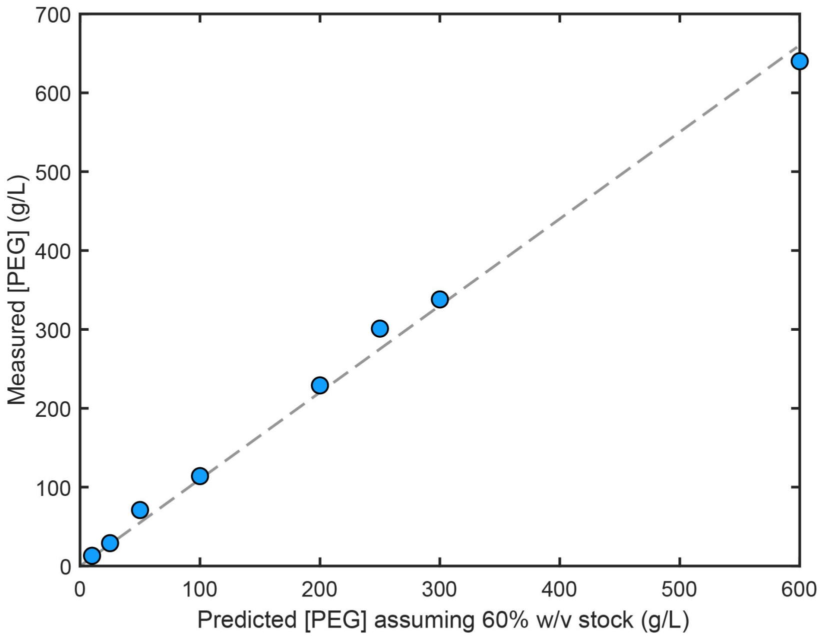

FIG. 7. **PEG calibration curve**
A representative calibration curve to determine the concentration of PEG stock solution. The measurements using ^{1}H NMR with DMF as concentration standard.
