# Supplementary material for: Sustained enzymatic activity and flow in crowded protein droplets
Source: Nat Commun. 2021 Nov 1;12:6293. doi: 10.1038/s41467-021-26532-0 (PMC8560906; doi:10.1038/s41467-021-26532-0)
Supplement: Supplementary file 5 — Description of Additional Supplementary Files [file 41467_2021_26532_MOESM5_ESM.pdf]

Title: Supplementary Movie 1.
Description: Flow inside the active droplets. The concentration of urease inside the droplets was 1.0 µM in presence of 100 mM urea. The fluorescent tracers (200 nm radius) are colored in white.

Title: Supplementary Data 1
Description: ^{1}H NMR spectra used for PEG concentration measurement for the droplets phase, supernatant phase and for the total composition (droplets and supernatant, overall suspension).
